# Temperature-Driven Activated Sludge Bacterial Community Assembly and Carbon Transformation Potential: A Case Study of Industrial Plants in the Yangtze River Delta

**DOI:** 10.3390/microorganisms12071454

**Published:** 2024-07-17

**Authors:** Qingsheng Xu, Yifan Jiang, Jin Wang, Rui Deng, Zhengbo Yue

**Affiliations:** 1 School of Resources and Environmental Engineering, Hefei University of Technology, Hefei 230009, China; 2018170627@mail.hfut.edu.cn (Q.X.); 2023010117@mail.hfut.edu.cn (Y.J.); sophiawj@hfut.edu.cn (J.W.); dengrui@hfut.edu.cn (R.D.); 2 Anhui Engineering Research Center of Industrial Wastewater Treatment and Resource Recovery, Hefei University of Technology, Hefei 230009, China; 3 Key Laboratory of Nanominerals and Pollution Control of Anhui Higher Education Institutes, Hefei University of Technology, Hefei 230009, China

**Keywords:** activated sludge, WWTPs, Temperature, microbial community, carbon transformation, CO_2_

## Abstract

Temperature plays a critical role in the efficiency and stability of industrial wastewater treatment plants (WWTPs). This study focuses on the effects of temperature on activated sludge (AS) communities within the A^2^O process of 19 industrial WWTPs in the Yangtze River Delta, a key industrial region in China. The investigation aims to understand how temperature influences AS community composition, functional assembly, and carbon transformation processes, including CO_2_ emission potential. Our findings reveal that increased operating temperatures lead to a decrease in alpha diversity, simplifying community structure and increasing modularity. Dominant species become more prevalent, with significant decreases in the relative abundance of *Chloroflexi* and *Actinobacteria*, and increases in *Bacteroidetes* and *Firmicutes*. Moreover, higher temperatures enhance the overall carbon conversion potential of AS, particularly boosting CO_2_ absorption in anaerobic conditions as the potential for CO_2_ emission during glycolysis and TCA cycles grows and diminishes, respectively. The study highlights that temperature is a major factor affecting microbial community characteristics and CO_2_ fluxes, with more pronounced effects observed in anaerobic sludge. This study provides valuable insights for maintaining stable A^2^O system operations, understanding carbon footprints, and improving COD removal efficiency in industrial WWTPs.

## 1. Introduction

Amidst escalating global water crises and pollution, studying wastewater treatment plants (WWTPs) becomes crucial given their central role in promoting water recycling and ecosystem preservation [1]. Temperature is a key environmental indicator that affects the treatment efficiency and stability of industrial wastewater treatment plants (WWTPs) [2]. On the one hand, in the process of industrial production, the wastewater generated often has a significantly different initial temperature due to different production processes [3]. On the other hand, factors such as seasonal changes can also lead to differences in wastewater temperature [4]. These wastewaters with different temperatures will enter various process sections of the sewage treatment plant, significantly affecting the operation of each process section.

Industrial wastewater contains a variety of pollutants—pharmaceuticals, pesticides, and xenobiotics among them [5,6]. These substances hinder the biochemical treatment in WWTPs, primarily due to diminished biodegradability [7,8]. This long-term industrial stress can shape relatively unique patterns of microbial communities [9,10]. However, there is a lack of research on the response of these special industrial microbial communities to different operating temperatures.

The A^2^O process is a widely used biochemical treatment technology in industrial WWTPs, with a high organic pollution treatment load and COD removal capacity [11]. The effective functional expression of activated sludge microbial communities (including bacteria and fungi) has contributed to the effective operation of the A^2^O process [12]. Some research results indicate that temperature significantly affects the performance and stability of the A^2^O process [13].

Temperature significantly affects the growth and functional assembly of microbial communities [14]. As the temperature increases, alpha diversity and network complexity decrease, while the process of de-community assembly gradually shifts from determinism to randomness [15,16]. However, in the A^2^O-activated sludge microbial community of industrial WWTPs, the microbial community subjected to industrial stress for a long time often exhibits stronger interspecific interaction and differentiated ecological niche distribution [17,18]. The influence mode of temperature on this industrial-activated sludge community still needs to be verified.

Temperature significantly affects the carbon conversion process in the A^2^O process [19,20]. On the one hand, temperature can affect the COD removal efficiency; on the other hand, it can also affect the potential for CO_2_ emissions [21]. Although the COD conversion ability of activated sludge is generally positively correlated with temperature, the cultivated activated sludge communities at different temperatures exhibit adaptability to different types of wastewater [22,23]. In many natural environments, temperature is the main driving factor affecting CO_2_ emissions from aquatic microbial communities [24,25,26]. However, in the A^2^O process of industrial WWTPs, the special activated sludge microbial community should exhibit a special temperature response pattern in carbon conversion. This process and mechanism still need further analysis.

The Yangtze River Delta region is one of China’s most critical industrial clusters, with solid representativeness among many industrial zones [27]. Therefore, AS from 19 industrial WWTPs A^2^O process units in the Yangtze River Delta region was taken as the research object to explore the effects of temperature on AS communities’ growth, functional assembly, and carbon transformation processes. In detail, the objectives of this study are to reveal, under different temperatures, (I) the composition and community evolution of AS communities in industrial WWTPs, (II) interactions and assembly patterns of microbial community functions in industrial WWTPs, and (III) the carbon conversion potential and CO_2_ emission potential of AS in industrial WWTPs.

## 2. Materials and Methods

### 2.1. Sampling Points and Collection Methods

**Figure 1:**
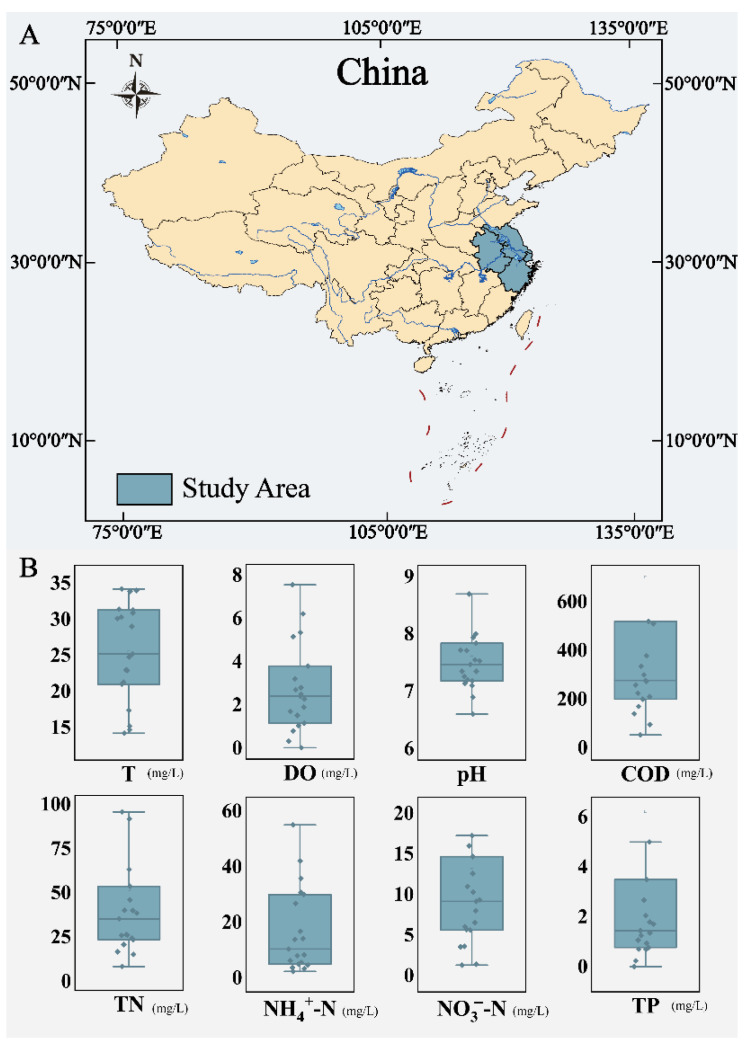
Study area and environmental indicators. (**A**) Geographical location of the study area. (**B**) Environmental variables of AS in each plant.

This study investigated AS from industrial WWTPs in the region of the Yangtze River Delta in China. A total of 38 AS samples were collected from 19 industrial WWTPs in the Zhejiang, Anhui, Jiangsu, and Shanghai regions (Figure 1A).

The influent of the selected WWTPs in this study contained industrial effluents from industries such as printing and dyeing, pharmaceuticals, pesticides, fine chemicals, and petrochemicals. The biochemical treatment modules in the WWTPs adopted the A^2^O treatment process, with the samples for this study collected from both anaerobic and oxic process units [28,29,30,31,32]. Detailed sample information is provided in the Supplementary Materials (Table S1).

In this study, the environmental temperature of the activated sludge samples showed significant inter-sample differences. Samples with higher temperatures can reach temperatures up to nearly 35 °C, while samples with lower temperatures can stabilize as low as below 15 °C. Using an average sample temperature of 25 °C as the dividing line, the samples in this study were categorized into the “high-temperature group” and “low-temperature group”. Further, the samples were subdivided into “high-temperature anaerobic group” (HA), “low-temperature anaerobic group” (LA), “high-temperature oxic group” (HO), and “low-temperature oxic group” (LO). In this study, the differences in other environmental variables such as DO and pH were relatively small, and the nutrients such as carbon and nitrogen were basically in the appropriate range for the growth and functioning of activated sludge, meeting the sample conditions for grouping research (Figure 1B) (Table S2).

### 2.2. Analytical Methods

For the on-site collected sludge samples, water temperature (T), pH, and dissolved oxygen (DO) were measured on-site using a portable water quality analyzer (MYRON L, 6PFC, San Diego, CA, USA) and a dissolved oxygen meter (HACH, HQ30d, Washington, DC, USA). Ammonia nitrogen (NH_4_^+^-N), nitrate nitrogen (NO_3_^−^-N), total nitrogen (TN), and chemical oxygen demand (COD) were measured according to the standard methods. Additionally, DNA was extracted from the samples using the FastDNA Spin Kit (Biomedicals, Santa Ana, CA, USA) reagent kit for microbial community analysis. The extracted DNA was amplified using 515F/806R primers targeting the V3-4 region of the 16S rRNA gene, and the amplified products were sequenced on the Illumina Miseq platform. Shanghai Pai Senno Biotechnology Co., Ltd. (Shanghai, China) provided sequencing services. For other sludge samples, environmental indicator data were obtained from corresponding publications for the publicly available database-collected sludge samples. The fastq files of the original sequences corresponding to the samples were obtained from the NCBI database. Microbial community analysis was conducted based on the corresponding primer sequences using the same denoising and calibration methods as the on-site samples. 

This study analyzed the characteristics and functions of activated sludge microbial communities from various research perspectives, such as alpha diversity, co-occurrence networks, Zi-Pi, random forest, linear regression, PICRUSt2, etc. Further details of methods can be found in the Supplementary Materials (Table S2). Although PICRUSt2 enables the prediction of functional potential within microbial communities, its reliance on 16S rRNA gene data introduces inherent limitations, especially concerning the absence of direct supporting evidence from metabolites or protein levels. Incorporating quantitative analysis in future studies holds promise to address this deficiency.

## 3. Results and Discussion

### 3.1. Microbial Community Evolution

In this study, the alpha index was used to characterize the microbial community of activated sludge, and Observed_species, Shannon, Faith_pd, and Pielou_e can characterize the richness, diversity, evolutionary diversity, and homogeneity of the microbial community, respectively.

In the high-temperature activated sludge sample group, microbial communities’ richness, diversity, and homogeneity are relatively low, at 22.79%, 11.18%, and 8.95%. This may be due to the selective pressure of temperature on microbial communities. Although some microorganisms metabolize faster at high temperatures, many are also sensitive to high temperatures, simplifying the microbial niche in the community [15]. At the same time, higher temperatures can also intensify the competition of microorganisms for substrates, allowing some microbial species to occupy a dominant position in this process, thereby occupying more ecological niches [33]. This phenomenon is more significant in anaerobic samples. In the high-temperature activated sludge sample group, the evolutionary diversity is relatively high at 9.95%. This may be because, under high-temperature conditions, some microorganisms in the microbial community with unique evolutionary characteristics are highlighted, often possessing specific functionalities, resulting in greater evolutionary differences in the community system [34]. Such an increase could indicate a reservoir of genetic novelty that might confer adaptive advantages under changing environmental conditions [35].

The composition of microbial communities in the four groups of sludge samples also showed significant differences. The relative abundance of *Chloroflexi* and *Actinobacteria* decreased significantly with increasing temperature, while the relative abundance of *Bacteroidetes* increased significantly. *Chloroflexi* is more sensitive to high temperatures, and metabolic activities and growth processes tend to be inhibited by high temperatures [36]. *Actinobacteria* also tend to be more common in low and mesophilic environments, where high temperatures may interfere with their enzymatic activities [37]. In contrast, *Bacteroidetes* tend to have a wider range of environmental requirements, and some strains have higher temperature adaptability, which may give them an advantage in the dynamics of activated sludge microbial communities [13]. This compositional shift may reflect a community-level adjustment towards organisms better suited for thermophilic conditions, influencing ecosystem services such as biodegradation efficiency. It is noteworthy that *Firmicutes* occupied a significant ecological niche in the high-temperature anaerobic sludge microbial community, partly because the phylum *Firmicutes* includes many species with strong metabolism and survival ability at high temperatures (e.g., *Bacillus* spp.); on the other hand, the *Firmicutes* phylum includes some species with a strong anaerobic metabolizing ability (e.g., *Clostridium* spp.) that can ferment and metabolize organic matter in anaerobic environments, producing short-chain fatty acids and other metabolites [33,38,39].

### 3.2. Microbial Community Functional Co-Occurrence

**Figure 2:**
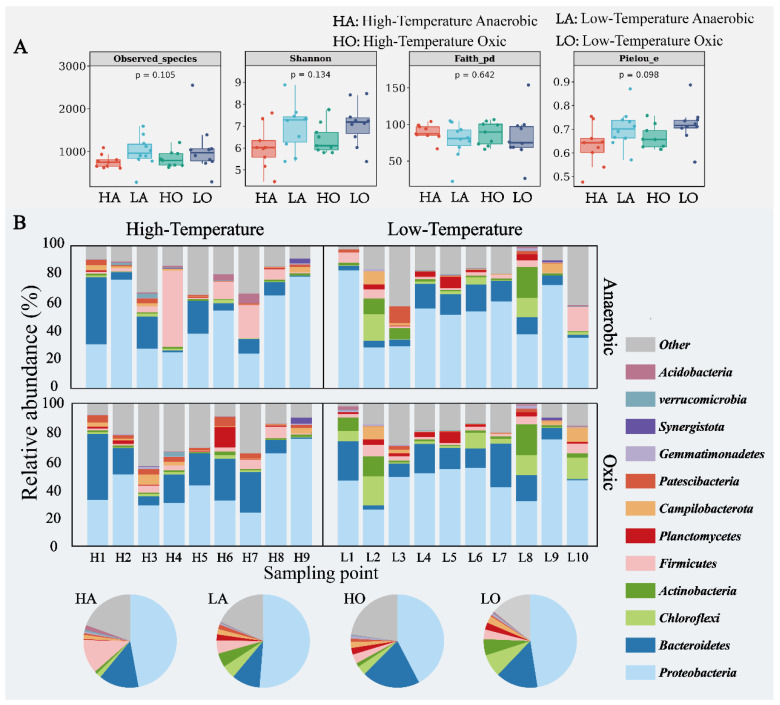
Microbial community characteristics of four types of AS in industrial WWTPs. (**A**) Alpha diversity index of the microbial community. (**B**) Microbial community composition at the level of phylum (the stack chart and pie chart represent the phylum composition of each sample and each sample group, respectively).

Co-occurrence network analysis (based on ASV) was performed on four groups of activated sludge samples and the relevant parameters of the network graph were calculated (Figure 2A,B). The co-occurrence networks of the four groups of activated sludge samples showed significant differences. Nodes reflected the effective functional ASVs in the network. In this study, the number of nodes in the high-temperature sample group was slightly lower than that in the low-temperature sample group, suggesting that the functional expressions in the sludge community were relatively more diverse as the temperature increased. Edges, degree, and graph density reflected the correlation strengths between the various correlation strengths between functional nodes. In this study, these parameters were significantly lower in the high-temperature sample group than in the low-temperature sample group, indicating that the correlation between the functional nodes of the sludge community was weaker as the temperature increased, and each function tended to be more independent. This phenomenon is more significant in oxic sludge. Modularity reflects the strength and clarity of subgroups in a network. In this study, the degree of modularity of the high-temperature sample group was significantly higher than that of the low-temperature sample group, indicating that with the increase in temperature, the different flora of the activated sludge microbial community tended to form relatively independent modules, and the interactions within the individual subgroups were enhanced.

These parameters suggest two distinct patterns of functional structure in activated sludge communities at high and low temperatures. Temperature exerts significant selective pressure on the structure and function of microbial communities. Elevated temperatures lead to a more modular and simplistic microbial community, and microbial communities showed more significant niche differentiation [40]. The selective pressure of higher temperatures also alters microbial interactions, allowing microbial populations adapted to higher temperatures to more fully utilize resources through competition, leading to a more simplified community structure [41,42].

**Figure 3:**
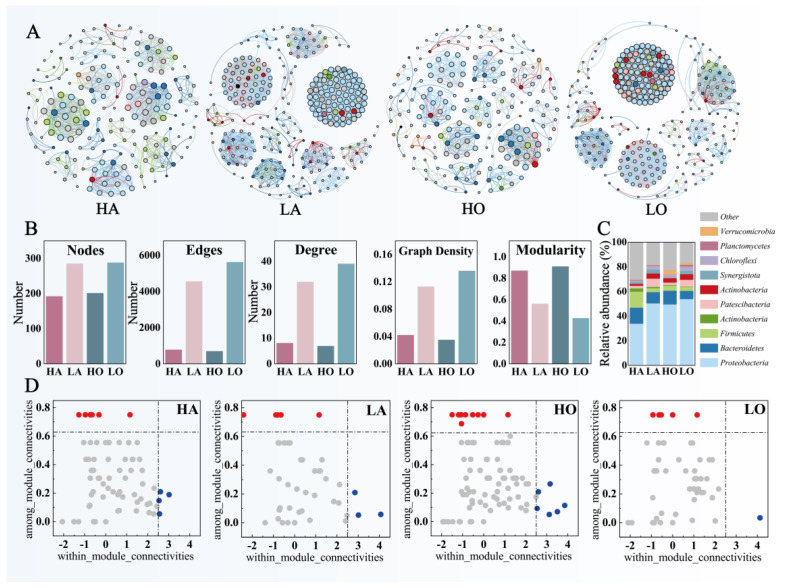
Functional expression of AS communities in four types of industrial WWTPs. (**A**) Microbial co-occurrence networks based on ASVs of different groups. The nodes are colored based on the phylum level of bacteria. A connection indicates a strong (Spearman’s ρ > 0.7) and significant (*p* < 0.01) correlation. (**B**) Comparison of the main parameters of the co-occurrence network. (**C**) Microbial community composition of co-occurrence network. (**D**) The roles of nodes in co-occurrence networks are shown by the distributions of their within-module connectivity (Zi) and among-module connectivity (Pi).

In addition, this study counted the phylum-level microbial taxonomic annotations for each ASV in the co-occurrence network (Figure 3C) to determine the relative contribution of each microbial phylum to the community-functional co-occurrence network. *Proteobacteria*, *Bacteroidetes*, and *Firmicutes* showed a more prominent role in the community-functional network. In contrast, the co-occurrence network of the high-temperature anaerobic sludge community showed a significant decrease in the function of *Proteobacteria* and a significant increase in the function of *Firmicutes*. This suggests that the effects on the acting microbial species differed significantly for both anaerobic and oxic sludge communities, even though higher temperatures caused similar changes in the co-occurrence network parameters [43]. Under the selective pressure of higher temperatures, *Firmicutes* showed more significant functionality in the anaerobic sludge community, playing a more important key microbial role [44,45].

Further, the within-module connectivity (Zi) and among-module connectivity (Pi) in the ASV functional co-occurrence network were calculated. The more nodes with Zi greater than 2.5 or Pi greater than 0.6, the higher the number of key functional microorganisms in the community. The number of key functional microorganisms in the community of the high-temperature sample group was significantly higher than that of the low-temperature sample group (Figure 3D). This also demonstrates that the simpler community structure expresses more functional microbial modules under the selective pressure of higher temperatures. More key microbial species expressed more significant and diverse functional roles in the community modules [46].

### 3.3. Microbial Community Carbon Fixation Potential

**Figure 4:**
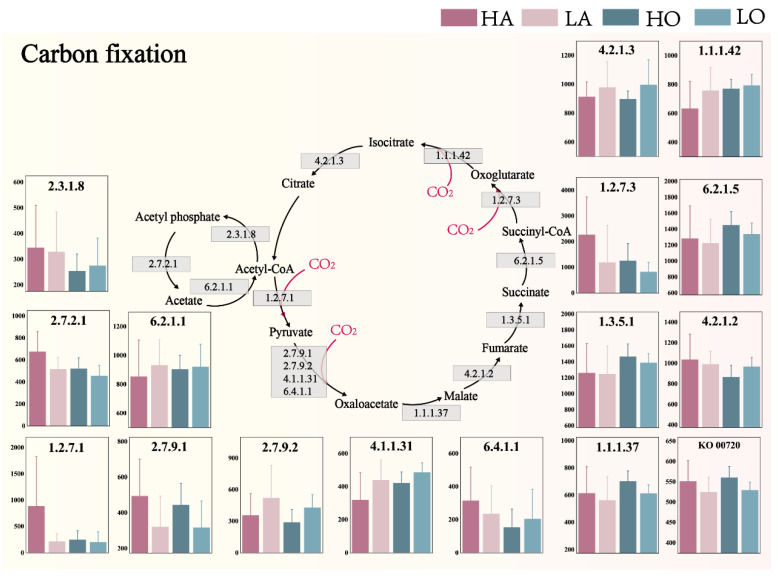
Carbon fixation transformation potential of different types of AS microbial communities in industrial WWTPs (Based on PICRUSt2 and KEGG, the subprocess is based on the numerical analysis of level 3 functional EC, and the overall process is based on the numerical analysis of level 2 functional KO) (KO00720 means a series of enzymes with the same pathway of “Carbon fixation pathways in prokaryotes”).

Based on the KEGG database, this study used PICRUSt2 to predict the carbon fixation transformation functions of the AS community (Figure 4). For the overall functioning of the activated sludge community, the potential for carbon fixation capacity was enhanced with increasing temperature (KO00720). This suggests that within a certain threshold of temperature change (15–35 °C), an increase in temperature enhances the potential for carbon fixation processes. This is usually associated with increased metabolic activity of carbon fixation-associated microorganisms, and their key enzyme activities, at higher temperatures [47,48].

Although the overall potential of the carbon fixation process is positively correlated with temperature, to investigate the CO_2_ fixation capacity of the activated sludge system in practical applications, several major CO_2_–substrate carbon conversion sub-processes were analyzed in detail. The differential response of these specific pathways to temperature fluctuations highlights the complexity of metabolic regulation in microbial communities and underscores the importance of considering multiple process levels when assessing ecosystem function under changing environmental conditions. These processes are “acetyl-CoA to pyruvate”, “pyruvate to oxaloacetate”, “succinyl-CoA to oxoglutarate”, and ‘oxoglutarate to isocitrate’. The potential of these processes directly reflects the ability of the activated sludge community to utilize CO_2_. Notably, the activated sludge in the high-temperature anaerobic group showed very significant potential enhancements in the conversion of acetyl-CoA to pyruvate (EC: 1.2.7.1) and the conversion of succinyl-CoA to oxoglutarate (EC: 1.2.7.3) by 309.05% and 90.69%. It is hypothesized that CoA may be one of the key factors in the enhanced carbon fixation capacity of the high-temperature anaerobic group. High temperature may increase the regeneration rate of CoA to ensure its availability in the corresponding metabolic pathway [49] and facilitate the rapid progression of acetyl-CoA and succinyl-CoA transformations [50,51]. As for the activated sludge microbial community, the enhancement of the above-mentioned relevant metabolic pathways is likely more significant under anaerobic conditions.

### 3.4. Microbial Community Glycolysis and TCA Cycle Potential

**Figure 5:**
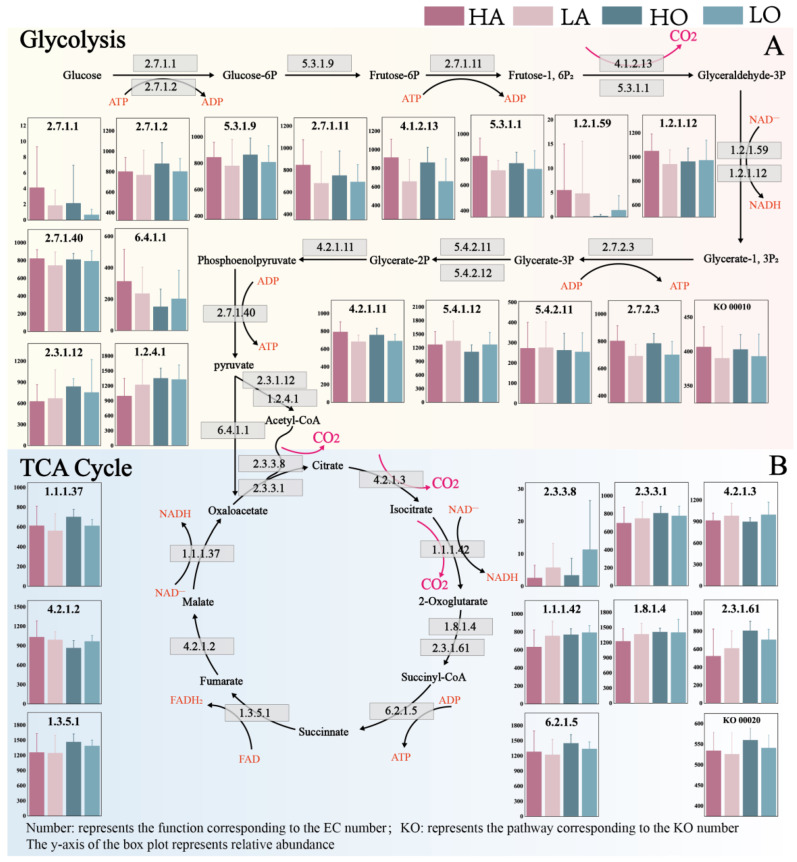
Carbon transformation potential of different types of AS microbial communities in industrial WWTPs (based on PICRUSt2 and KEGG, the subprocess is based on the numerical analysis of level 3 functional EC, and the overall process is based on the numerical analysis of level 2 functional KO). (**A**) Glycolysis process. (**B**) TCA cycle process (KO00010 and KO00020 mean series of enzymes with the same pathway of “Glycolysis/Gluconeogenesis” and “TCA cycle”).

Based on the KEGG database, this study used PICRUSt2 to predict the glycolysis and TCA cycle transformation functions of the AS community (Figure 5). For the overall functioning of the activated sludge community, the potential for glycolysis and TCA cycle was enhanced with increasing temperature (KO00010, KO00020). This indicates that within a certain threshold of temperature change (15–35 °C), the increase in temperature enhanced the potential of glycolysis and TCA cycling. This is consistent with the trend of enhanced microbial metabolic activity and associated enzyme activities at higher temperatures [52]. Notably, such temperature-mediated boosts could reflect an adaptive mechanism by which microorganisms optimize energy harvest under dynamic environmental conditions. In comparison, the enhancement of the overall potential of glycolysis was more pronounced in anaerobic-activated sludge and the enhancement of the overall potential of the TCA cycle was more pronounced in oxic-activated sludge. This may be because glycolysis under an anaerobic environment is the main pathway for microorganisms to obtain energy, and the increase in temperature selectively promoted the growth and metabolic activity of glycolytic anaerobic microorganisms [53]. The TCA cycle is the main mode of energy metabolism in microorganisms in oxic environments, and elevated temperatures may be able to increase the activities of related enzymes and electron transfer rates [54]. Thus, the temperature sensitivity of these pathways underscores the intricate balance between metabolic efficiency and environmental adaptability in activated sludge communities.

Further, the conversion processes of CO_2_ output in glycolysis and TCA cycle were analyzed in detail. In glycolysis, CO_2_ was mainly produced in the conversion of “Frutose-1, 6P2 to Glyceraldehyde-3P” (EC:4.1.2.3, EC:5.3.1.1). For both anaerobic and oxic sludge, the transformation potential of the high-temperature sample group was 20.5% and 16.8% higher than that of the low-temperature sample group. This indicates that the increase in temperature directly promotes the CO_2_ emission potential* of the glycolysis process. In the TCA cycle, CO_2_ is mainly produced in three conversion processes: “acetyl-CoA to citrate”, “citrate to isocitrate”, and “isocitrate to 2-oxoglutarate”. For both anaerobic and oxic sludge, the conversion potential of the high-temperature sample group was 11.1% and 7.1% lower than that of the low-temperature sample group. This suggests that the increase in temperature, while promoting the potential of the TCA cycle, inhibited the production of CO_2_. It is hypothesized that high temperatures may activate other metabolic pathways parallel to or intersecting with the TCA cycle, allowing a portion of CO_2_-associated intermediates to be directed to other pathways or to be used in synthesizing cells [55,56].

### 3.5. Microbial Community Contribution by Indicators

**Figure 6:**
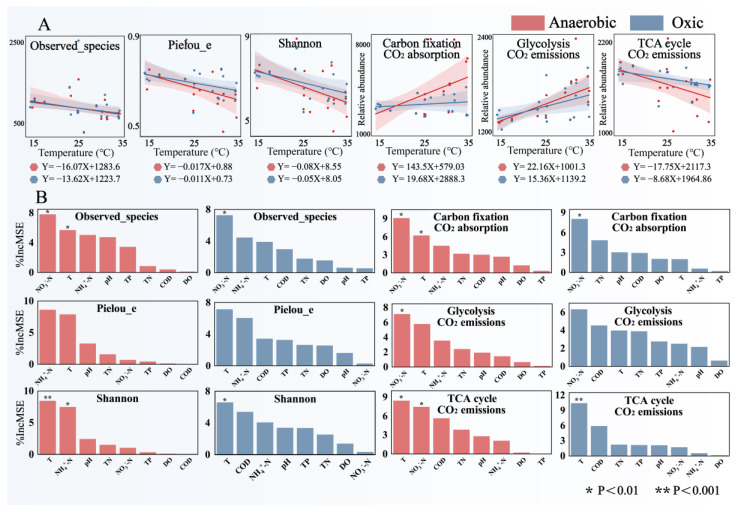
Contribution of environmental indicators to the community characteristics and CO_2_ absorption and emissions potential of anaerobic and oxic AS in industrial WWTPs (based on alpha diversity and microbial functional potential predicted by PICRUSts2). (**A**) Construction of diversity index linear regression model to validate the effect of temperature. (**B**) Contribution of environmental indicators to sludge microbial community characteristics analyzed using random forest.

Two types of samples, anaerobic-activated sludge and oxic-activated sludge, were analyzed using statistical methods to explore whether temperature could significantly explain the differences between the two groups of samples. Changes in community characteristics and CO_2_ absorption and emission potential of the sludge microbial community with temperature changes were analyzed using linear regression methods (Figure 6A). It can be seen that the alpha diversity of microbial communities gradually decreased with increasing temperature. This indicates that the increase in temperature enhanced the community competition and a few dominant populations played a greater systematic role [57]. The potential for CO_2_ absorption during carbon fixation increased significantly with increasing temperature in anaerobic sludge, whereas this was not evident in oxic sludge. This indicates that the carbon fixation pathway was enhanced in anaerobic sludge with increasing temperature, which significantly increased the CO_2_ absorption potential, suggesting a selective advantage for carbon-conserving strategies under thermal enrichment, whereas the contribution of this pathway was smaller in the oxic environment [58]. With increasing temperature, the CO_2_ emission potential during glycolysis grew, while the CO_2_ emission potential decreased in the TCA cycle, both phenomena being more pronounced in anaerobic sludge. This is consistent with the findings of the previous study (Figure 5) and is related to the adjustment of microbial metabolic properties and changes in microbial community structure, underscoring the intricate interplay between temperature, metabolic pathways, and community dynamics. The extent to which each environmental indicator explained the differences in community characteristics and CO_2_ absorption and emission potential of the sludge microbial community was verified using the random forest approach (Figure 6B). It can be seen that temperature more significantly explained the community differences between samples. For CO_2_ absorption and release potential, temperature had a higher weighting of the degree of explanation for anaerobic sludge, which is also consistent with previous findings, reaffirming temperature’s pivotal role in shaping anaerobic sludge’s CO_2_ metabolism.

## 4. Conclusions

This study investigated the response patterns of A^2^O-activated sludge microorganisms in 19 industrial WWTPs in the Yangtze River Delta region at different temperatures. This has guiding significance for maintaining the stable operation of A^2^O sludge systems in sewage treatment plants, understanding the carbon footprint during the process, and improving COD removal efficiency.

- As the operating temperature increases, the alpha diversity of A^2^O-activated sludge decreases, the community structure becomes simpler and more modular, and a few dominant species occupy more ecological niches. The relative abundance of *Chloroflexi* and *Actinobacteria* significantly decreased, while the relative abundance of *Bacteroidetes* and *Firmicutes* increased. *Firmicutes* exhibit high-temperature adaptability in anaerobic sludge communities.
- As the operating temperature increases, the carbon conversion potential of A^2^O-activated sludge increases overall. The absorption potential of CO_2_ is enhanced, especially in anaerobic sludge. During glycolysis and TCA cycle, the potential for CO_2_ release increases and decreases, respectively. Upcoming studies should focus on analyzing transport and primary metabolism-related proteins that can directly illuminate links between carbon uptake and biomass synthesis. This will not only empirically validate the study’s conclusions but also enhance our understanding of temperature-driven impacts on activated sludge microbial community metabolism.
- Temperature is an important factor in explaining the characteristics of microbial communities and the differences in CO_2_ absorption and emission potential. In terms of the absorption and emission potential of CO_2_, the influence of temperature on anaerobic sludge is more significant.

## Data Availability

The raw data supporting the conclusions of this article will be made available by the authors on request.

## Supplementary Materials

The following supporting information can be downloaded at: https://www.mdpi.com/article/10.3390/microorganisms12071454/s1.
