# Supplementary material for: Temperature-Driven Activated Sludge Bacterial Community Assembly and Carbon Transformation Potential: A Case Study of Industrial Plants in the Yangtze River Delta
Source: Microorganisms. 2024 Jul 17;12(7):1454. doi: 10.3390/microorganisms12071454 (PMC11278906; doi:10.3390/microorganisms12071454)
Supplement: Supplementary file 1 [file microorganisms-12-01454-s001.zip › microorganisms-3092217-supplementary.pdf]

# Supplementary Material

# Temperature-driven activated sludge community assembly and carbon transformation potential: a case study of industrial plants in the Yangtze River Delta

**Qingsheng Xu [a,b,#], Yifan Jiang[a,#], Jin Wang [a,b,c], Rui Deng [a,b], Ding Ma [a,b], Zhengbo Yue [a,b,c,*]**

[a] School of Resources and Environmental Engineering, Hefei University of Technology, Hefei, Anhui 230009, China

[b] Anhui Engineering Research Center of Industrial Wastewater Treatment and Resource Recovery, Hefei University of Technology, Hefei, Anhui 230009, China

[c] Key Laboratory of Nanominerals and Pollution Control of Anhui Higher Education Institutes, Hefei University of Technology, Hefei, Anhui 230009, China

## 1. Activated sludge sample information

**Tab.S1** Activated sludge sample information

| Sample number | WWTP | Dissolved oxygen | Location | Data Sources | NCBI number | References |
|---|---|---|---|---|---|---|
| 1 | AH1 | oxic | Anhui | sampling | PRJNA 1045802 | - |
| 2 | | anoxic | | | | |
| 3 | AH2 | oxic | | | | |
| 4 | | anoxic | | | | |
| 5 | AH3 | oxic | | NCBI | PRJNA 547875 | doi:10.1016/j.scitotenv.2019.06.432[1] |
| 6 | | anoxic | | | | |
| 7 | ZJ1 | oxic | Zhejiang | | PRJNA 803938 | doi:10.1016/j.envint.2022.107486[2] |
| 8 | | anoxic | | | | |
| 9 | ZJ2 | oxic | | | | |
| 10 | | anoxic | | | | |
| 11 | ZJ3 | oxic | | | | |
| 12 | | anoxic | | | | |
| 13 | ZJ4 | oxic | | | | |
| 14 | | anoxic | | | | |
| 15 | ZJ5 | oxic | | | | |
| 16 | | anoxic | | | | |
| 17 | ZJ6 | oxic | | | | |
| 18 | | anoxic | | | | |
| 19 | ZJ7 | oxic | | | | |
| 20 | | anoxic | | | | |
| 21 | ZJ8 | oxic | | | | |
| 22 | | anoxic | | | | |
| 23 | ZJ9 | oxic | | | | |
| 24 | | anoxic | | | | |
| 25 | ZJ10 | oxic | | | | |
| 26 | | anoxic | | | | |
| 27 | ZJ11 | oxic | | | | |
| 28 | | anoxic | | | | |
| 29 | SH1 | oxic | Shanghai | | PRJNA 274970 | doi:10.1007/s00253-016-7307-0[3] |
| 30 | | anoxic | | | | |
| 31 | SH2 | oxic | | | | |
| 32 | | anoxic | | | | |
| 33 | SH3 | oxic | | | | |
| 34 | | anoxic | | | | |
| 35 | SH4 | oxic | | | | |
| 38 | | anoxic | | | | |
| 37 | JS | oxic | Jiangsu | | PRJNA 489993 | doi:10.1016/j.cej.2018.11.167[4] |
| 38 | | anoxic | | | | |

**Tab.S2** Activated sludge sample information

| WWTP | COD (mg/L) | TN (mg/L) | $NH_4^+$-N (mg/L) | $NO_3^-$-N (mg/L) | TP (mg/L) | pH | DO (mg/L) | T (°C) |
|---|---|---|---|---|---|---|---|---|
| AH1 | 1800 | 95.7 | 55 | 32 | 24 | 10.5 | 0.8 | 23 |
| AH2 | 200 | 26.4 | 8 | 16 | 18 | 8.7 | 1.52 | 25 |
| AH3 | 53 | 20.775 | 13.85 | 5.6785 | 1.45 | 7.71 | 3.2 | 23.3 |
| ZJ1 | 510 | 38.56 | 4.6 | 9.33 | 1.71 | 7.53 | 3.8 | 34 |
| ZJ2 | 258 | 35.28 | 6.29 | 1.29 | 0.24 | 7.35 | 1.7 | 31.4 |
| ZJ3 | 1220 | 295.41 | 5.62 | 10.28 | 51.04 | 7.94 | 0.02 | 33.9 |
| ZJ4 | 300 | 26.08 | 4.96 | 5.62 | 2.67 | 7.47 | 1.05 | 24.9 |
| ZJ5 | 224 | 25.87 | 3.7 | 3.6 | 1.07 | 7.26 | 7.55 | 30.3 |
| ZJ6 | 1060 | 91.7 | 16.8 | 74.9 | 0.7 | 7.18 | 5.34 | 24.8 |
| ZJ7 | 210 | 23.64 | 8.34 | 9.14 | 1.8 | 7.84 | 1.15 | 30.9 |
| ZJ8 | 170 | 8.41 | 2.38 | 6.03 | 0.71 | 7.14 | 5.16 | 34.2 |
| ZJ9 | 140 | 16.76 | 10.41 | 3.55 | 0.01 | 7.72 | 0.32 | 30.1 |
| ZJ10 | 520 | 24.36 | 14.16 | 6.54 | 0.78 | 7.55 | 6.21 | 29 |
| ZJ11 | 95 | 15.24 | 3.42 | 1.41 | 1.36 | 7.35 | 2.28 | 31.3 |
| SH1 | 335 | 40.2 | 26.8 | 10.988 | 1.25 | 7.2 | 2.7 | 14.2 |
| SH2 | 277 | 63.15 | 42.1 | 17.261 | 2.05 | 7.1 | 2.4 | 17.4 |
| SH3 | 378 | 53.7 | 35.8 | 14.678 | 0.95 | 6.6 | 2.5 | 15.2 |
| SH4 | 273 | 46.05 | 30.7 | 12.587 | 3.5 | 6.9 | 2.8 | 14.7 |
| JS1 | 5300 | 40 | 30 | 8 | 5 | 8 | 1.9 | 26.2 |

## 2. 16s rRNA test means and other analytical methods

In this study, the DADA2 method was employed for primer trimming, quality filtering, denoising, merging, and chimera removal to generate unique sequences referred to as amplicon sequence variants (ASVs), which were further represented as ASV feature tables to depict their abundances in the samples. Following the length trimming of sequences in the table, rarefaction was performed using QIIME2 (2019.4) at a depth set to 95% of the minimum sample sequence count.

The sequence data was aligned with the Greengenes database (Release 13.8, http://greengenes.secondgenome.com/) to obtain the annotation information at various taxonomic levels, enabling taxonomical composition analysis of the samples.

In this study, PICRUSt2 (Phylogenetic Investigation of Communities by Reconstruction of Unobserved States) was employed to align the 16S rRNA feature sequences with reference sequences, construct an evolutionary tree, and utilize the Castor hidden state prediction algorithm. Based on the gene family copy numbers corresponding to the reference sequences in the evolutionary tree, the closest sequence species of the feature sequences were inferred to obtain their gene family copy numbers. Integrating the abundance of feature sequences in each sample, the gene family copy numbers of each sample were computed and mapped to the KEGG (https://metacyc.org/) database to obtain the abundance data of metabolic pathways in each sample.

Based on the rarefied ASV table, point and edge files were generated using R 4.3.1 and the packages "Hmisc" and "igraph" for co-occurrence network analysis. The network graph was visualized using Gephi 0.9.2, with coloring at the phylum level, and the topological properties of the network were calculated, including the number of nodes, edges, degree, graph density, connected components, to evaluate the structural characteristics of the network. The more links formed between nodes, the

closer their positions in the network. The size of nodes represents the number of connections (degree); larger nodes indicate a greater number of connections with other species. Roles of nodes in co-occurrence networks shown by the distributions of their within-module connectivity (Zi) and among-module connectivity (Pi), Based on the main ASV, R 4.3.1 was used to screen out suitable samples and plot (r>0.8).

The alpha diversity is an indicator of the richness, diversity and evenness of species in a localized homogeneous habitat, also known as within-habitat diversity. In order to have a more comprehensive assessment of the alpha diversity of the microbial community, the present study characterized richness by the Chao1 and Observed species indices, diversity by the Shannon indices, and evenness by the Pielou's evenness index to characterize evenness. Using the unleveled ASV table, the command "qiime diversity alpha-rarefaction" was invoked with the parameters "--p-steps 10 --p-min-depth 10 --p-iterations 10", i.e., the minimum leveling depth is 10, and the parameter "--p-max-depth" is set to 95% of the minimum sequencing depth of all the samples, and then 10 depths are selected evenly between this depth and the minimum depth, and each depth value is leveled 10 times, and the alpha diversity index of the selected samples is calculated. alpha diversity index. The average of the scores at the maximum leveling depth was chosen as the alpha diversity index. The above data was plotted as a box plot using R Studio script to visualize the difference in alpha diversity between the two groups of samples, and the significance of the difference could be verified by using the Kruskal-Wallis rank sum test and dunn'test as a post hoc test. Detailed calculations of the indicators can be found on the web site URL: http://scikit-bio.org/docs/latest/generated/skbio.diversity.alpha.html#module-skbio.diversity.alpha for details.

Random forest analysis is an analytical method of assessing the importance of variables using a

decision tree approach. In this study, three random forest models were explored separately. (I) the extent to each environmental variables contributed to community alpha diversity; (II) the extent to each environmental variables contributed to carbon transformation potential; In this study, Random Forest analysis was conducted using three R packages: "randomForest", "rfPermute", and "ggplot2". Conducted random forests 1000 times. The final results were generated through a three-step process involving extracting predictor explanatory rates, assessing the significance of predictor variables, and merging and sorting the table of explanatory rates for significant factors. The ranking responds to the degree of influence of each variable on carbon transformation potential and community diversity, with higher %IncMSE values indicating greater influence. A negative %IncMSE indicates that the factor has no significant effect on the variable under study.

Redundancy Analysis (RDA) is a powerful multivariate statistical technique used to explore and elucidate the relationships between response variables and explanatory variables. It accomplishes this by generating new dimensions through linear combinations of explanatory variables, thereby maximizing the capacity to explain variations in response variables. This analytical approach aids in comprehending how explanatory variables impact response variables, all while taking into account inter-variable correlations. In our study, RDA redundancy analysis was performed utilizing the relative abundance of microorganisms (phylum level) and physicochemical indicators. Each data point within the analysis represents an individual sample, and samples belonging to different groups are distinguished by various colors. The proximity of two data points in the graphical representation indicates a higher degree of similarity between the respective samples. Furthermore, the angle formed by the arrows in the analysis reflects the strength of correlation, with acute angles denoting a positive correlation and obtuse angles indicating a negative correlation. The length of the radii, on the other

hand, serves as an indicator of the magnitude of factors' influence on the system. The RDA analysis was performed by the genescloud tools (https://www.genescloud.cn). P-values above the ordination diagram represent P-values obtained using the permutation test random permutation nonparametric test; the smaller the P-value, the more significant the effect of the influencing factor on colony ASV abundance. Percentages in parentheses on the axes represent the proportion of the variance in the raw data that can be explained by the corresponding axes.

The various statistical methods used in this study are shown in the table below (Tab.S3):

**Tab.S3** Summary of statistical methods used in this study

| Figure | Analytical method | Explain |
| --- | --- | --- |
| Fig.2 A | Alpha indexes | The alpha diversity is an indicator of the richness, diversity and evenness of species in a localized homogeneous habitat, also known as within-habitat diversity.<br>In order to have a more comprehensive assessment of the alpha diversity of the microbial community, the present study characterized richness by the Chao1 and Observed species indices, diversity by the Shannon indices, and evenness by the Pielou's evenness index to characterize evenness. |
| Fig.3 A-C | Co-occurrence network | Based on the rarefied ASV table, point and edge files were generated using R 4.3.0 and the packages "Hmisc" and "igraph" for co-occurrence network analysis. The network graph was visualized using Gephi 0.9.2, with coloring at the phylum level, and the topological properties of the network were calculated, including the number of nodes, clustering coefficient, average path length, network diameter, and density, to evaluate the structural characteristics of the network. The more links formed between nodes, the closer their positions in the network. The size of nodes represents the number of connections (degree); larger nodes indicate a greater number of connections with other species.<br>Every connection indicates a strong (Spearman's $\rho > 0.7$) and significant ($p< 0.01$) correlation. |
| Fig.3 D | ZI-PI | The modular analysis of community function co-occurrence network was carried out, and the nodes with high modularity ($r>0.8$) were selected.<br>The within-module connectivity (Zi) and among-module connectivity (Pi) were calculated. Zi measures the connectivity between nodes in the same module in the network. Pi measures the degree of connectivity between different modules. When $Zi > 2.5$, we believe that the node has significant connectivity within the module. When $Pi > 0.6$, we believe that the node has significant connectivity between modules. |

| Figure | Analytical method | Explain |
| --- | --- | --- |
| Fig.6 B | Random forest | Random forest analysis is an analytical method of assessing the importance of variables using a decision tree approach.In this study, Random Forest analysis was conducted using three R packages: "randomForest", "rfPermute", and "ggplot2".<br>Conducted random forests 1000 times. The final results were generated through a three-step process involving extracting predictor explanatory rates, assessing the significance of predictor variables, and merging and sorting the table of explanatory rates for significant factors.<br>The ranking responds to the degree of influence of each variable on carbon transformation potential ($CO_2$ absorption and emission) and community diversity, with higher %IncMSE values indicating greater influence.<br>(The random forest itself does not provide the confidence interval like the traditional statistical model) |
| Fig.6 A | Linear regression | Linear regression is a statistical method used to model and analyze the relationship between two or more variables. Specifically, the linear regression model assumes that there is a linear relationship between the response variable and one or more explanatory variables, and verifies it.<br>The shaded part is the 95% confidence interval of the linear regression model. |

| Figure | Analytical method | Explain |
| --- | --- | --- |
| Fig.4-5 | PICRUSt2 | In this study, PICRUSt2 (Phylogenetic Investigation of Communities by Reconstruction of Unobserved States) was employed to align the 16S rRNA feature sequences with reference sequences, construct an evolutionary tree, and utilize the Castor hidden state prediction algorithm. Based on the gene family copy numbers corresponding to the reference sequences in the evolutionary tree, the closest sequence species of the feature sequences were inferred to obtain their gene family copy numbers. Integrating the abundance of feature sequences in each sample, the gene family copy numbers of each sample were computed and mapped to the KEGG (https://metacyc.org/) database to obtain the abundance data of metabolic pathways in each sample. By predicting and analyzing the secondary function (KO) and tertiary function (EC) of microorganisms, the potential of carbon transformation function was obtained. The histogram was drawn by calculating the average and variance of each group of samples. |

## Reference

[1] L. Zhang, Z. Shen, W. Fang, G. Gao, Composition of bacterial communities in municipal wastewater treatment plant, Science of The Total Environment, 689 (2019) 1181-1191.

[2] W. Chen, J. Wei, Z. Su, L. Wu, M. Liu, X. Huang, P. Yao, D. Wen, Deterministic mechanisms drive bacterial communities assembly in industrial wastewater treatment system, Environment International, 168 (2022) 107486.

[3] P. Gao, W. Xu, P. Sontag, X. Li, G. Xue, T. Liu, W. Sun, Correlating microbial community compositions with environmental factors in activated sludge from four full-scale municipal wastewater treatment plants in Shanghai, China, Applied Microbiology and Biotechnology, 100 (2016) 4663-4673.

[4] J. Liang, W. Mai, J. Tang, Y. Wei, Highly effective treatment of petrochemical wastewater by a super-sized industrial scale plant with expanded granular sludge bed bioreactor and aerobic activated sludge, Chemical Engineering Journal, 360 (2019) 15-23.
